# Patient contrastive learning: A performant, expressive, and practical approach to electrocardiogram modeling

**DOI:** 10.1371/journal.pcbi.1009862

**Published:** 2022-02-14

**Authors:** Nathaniel Diamant, Erik Reinertsen, Steven Song, Aaron D. Aguirre, Collin M. Stultz, Puneet Batra

**Affiliations:** 1 Research Laboratory of Electronics, MIT, Cambridge, Massachusetts, United States of America; 2 Data Sciences Platform, Broad Institute of MIT and Harvard, Cambridge, Massachusetts, United States of America; 3 Division of Cardiology, Massachusetts General Hospital, Boston, Massachusetts, United States of America; 4 Center for Systems Biology, Massachusetts General Hospital Research Institute and Harvard Medical School, Boston, Massachusetts, United States of America; 5 Wellman Center for Photomedicine, Massachusetts General Hospital Research Institute and Harvard Medical School, Boston, Massachusetts, United States of America; 6 Department of Electrical Engineering and Computer Science, MIT, Cambridge, Massachusetts, United States of America; 7 Harvard-MIT Division of Health Sciences and Technology, Cambridge, Massachusetts, United States of America; University of Zurich, SWITZERLAND

## Abstract

Supervised machine learning applications in health care are often limited due to a scarcity of labeled training data. To mitigate the effect of small sample size, we introduce a pre-training approach, **P**atient **C**ontrastive **L**earning of **R**epresentations (PCLR), which creates latent representations of electrocardiograms (ECGs) from a large number of unlabeled examples using contrastive learning. The resulting representations are expressive, performant, and practical across a wide spectrum of clinical tasks. We develop PCLR using a large health care system with over 3.2 million 12-lead ECGs and demonstrate that training linear models on PCLR representations achieves a 51% performance increase, on average, over six training set sizes and four tasks (sex classification, age regression, and the detection of left ventricular hypertrophy and atrial fibrillation), relative to training neural network models from scratch. We also compared PCLR to three other ECG pre-training approaches (supervised pre-training, unsupervised pre-training with an autoencoder, and pre-training using a contrastive multi ECG-segment approach), and show significant performance benefits in three out of four tasks. We found an average performance benefit of 47% over the other models and an average of a 9% performance benefit compared to best model for each task. We release PCLR to enable others to extract ECG representations at https://github.com/broadinstitute/ml4h/tree/master/model_zoo/PCLR.

## Author summary

ECGs are a rich source of cardiac health information. Many recent works have shown that deep learning can extract new information from ECGs when there are a sufficient number of labeled data. However, when there are not enough labeled data or a clinician scientist does not have the resources to train a deep learning model from scratch, options are limited. We introduce Patient Contrastive Learning of Representations (PCLR), an approach to train a neural network that extracts representations of ECGs. The only labels required to train PCLR are which ECG comes from which patient. The resulting ECG representations can be used directly in linear models for new tasks without needing to finetune the neural network. We show PCLR is better than using a set of handpicked features for four tasks, and better than three other deep learning approaches for three out of four tasks evaluated. Furthermore, PCLR is better than training a neural network from scratch when training data are limited. PCLR is one of the first attempts at releasing and evaluating a pre-trained ECG model with the purpose of accelerating deep learning ECG research.

This is a *PLOS Computational Biology* Methods paper.

## Introduction

Scarcity of labeled training data prevents the full clinical impact of supervised machine learning in health care. As one example, sudden cardiac death (SCD) kills over 450,000 Americans per year [1], yet large observational datasets, which contain millions of patient records, typically only have data for a small number of SCDs. Unlike machine learning applications outside of health care, it is not routinely possible to significantly increase the number of cases by labeling more data points because the prevalence of the disorder of interest is often very low. The resulting lack of statistical power is a significant impediment to the development of accurate risk models [2].

An approach that has proven successful when there are few labeled training examples for a given task of interest is pre-training: neural networks are first trained on a large corpus of data for a set of related tasks, and then the pre-trained models are fine-tuned on the task of interest. Pre-training allows models to learn from large datasets to improve performance on smaller, potentially more important datasets. There are many explanations for why pre-training improves performance in small datasets, including pre-training acting as a regularization [3] and pre-training as learning salient features [4]. Pre-training has proven successful across many domains, including health care [5]. The ideal pre-training strategy is:

- Performant: it maximizes performance on limited training data
- Expressive: it can be used to develop models for multiple tasks
- Practical: it is easy-to-use for those unfamiliar with deep learning

**Fig 1:**
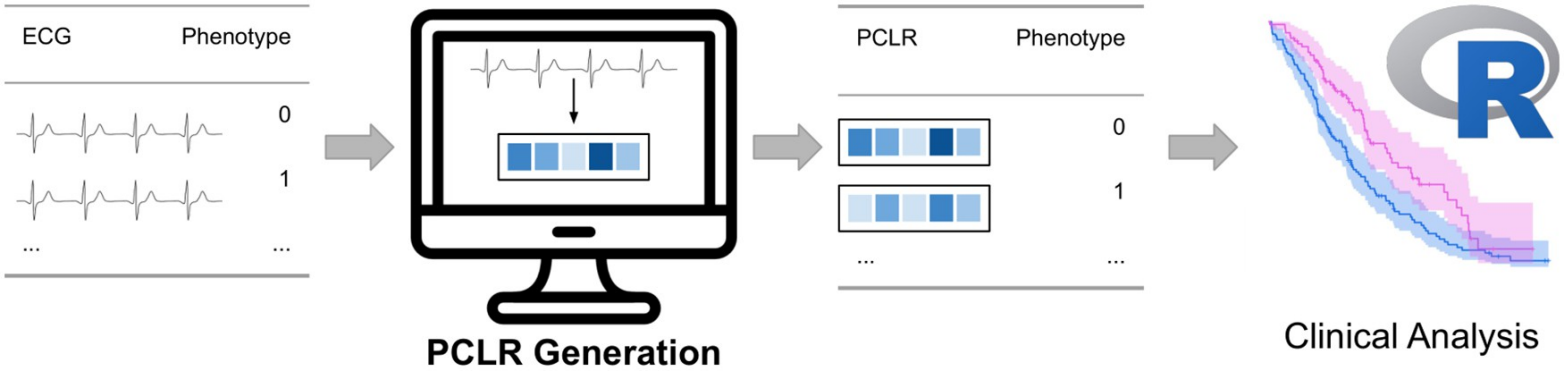
Example workflow using PCLR. A clinical researcher uploads ECGs to a service which returns a PCLR representation for each ECG. The representations can then be used by the clinician in models standard to clinical research workflows, including, for example, Cox proportional hazards models.

In the past, pre-training strategies in health-care have focused on the first goal, with a few notable exceptions that also consider the third goal [6]. As larger groups of clinical researchers adopt machine learning approaches to more tasks, expressivity and ease-of-use have become more critical. Clinical scientists often do not have the resources, or expertise, needed to retrain deep learning models for their specific task, although they do have the greatest insight into model deployment needs. We therefore develop and validate Patient Contrastive Learning of Representations (PCLR) for 12-lead ECGs to satisfy all these objectives. We demonstrate the effectiveness of PCLR using two large hospital system data sets over four distinct ECG tasks. PCLR yields feature vectors optimized for *linear* models, making them easily used by clinical researchers out-of-the-box (Fig 1).

How does PCLR do this? PCLR is a uniquely clinically-driven modification of SimCLR [7]: a contrastive learning approach that builds expressive representations of high-resolution data. However, where SimCLR is graded on whether a model can resolve artificial data augmentations, PCLR is graded on whether it can resolve patient identity over time. PCLR maps ECGs acquired at different times from a given patient to the same region within a contrastive latent space. The success of PCLR compared to disease-specific approaches (see Results) shows that patient-centric latent representations are a new direction for deep learning research deserving attention. As Sir William Osler said, “The good physician treats the disease; the great physician treats the patient who has the disease.” PCLR is a step for deep learning towards Osler’s dictum.

### Brief ECG background

**Fig 2:**
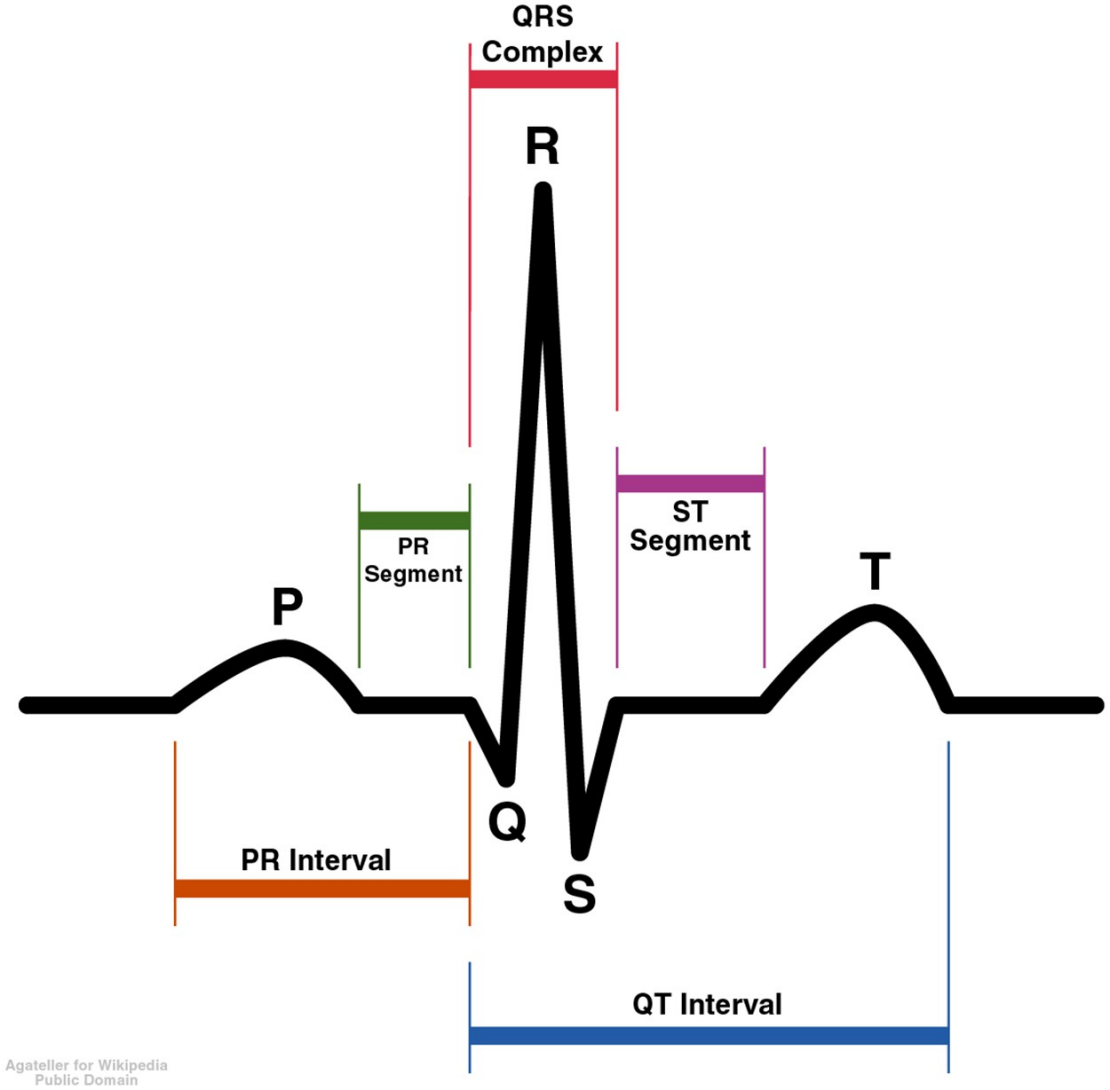
Idealized ECG heartbeat with waves labeled.

The ECG measures the electrical activity of the heart and is one of the cardiologist’s oldest tools. An ECG is typically recorded using 10 different electrodes placed at different parts of the body. The voltage difference between 12 different combinations of the electrodes is typically measured for 10 seconds. The 12 voltage difference measurements are called ECG leads. Five different waves are commonly noted in each healthy heartbeat, which are labeled P, Q, R, S, and T (Fig 2). The duration and maximum height of each peak can be measured, and each has different clinical implications.

### Related work

#### Deep learning on ECGs

There is a growing body of work that applies techniques from deep image classification to 12-lead ECGs in the presence of large labeled datasets. [8] train a residual network in a cohort containing millions of labeled ECGs to classify cardiac blocks and other arrhythmias with high accuracy, and we use their model as a baseline for comparison. Residual networks have also been shown to outperform automatic labeling systems [9], and even physician labels [10], and to triage patients [11]. Latent features of the ECG have also been shown to be useful for a wide range of tasks, such as to regress age from the ECG as a marker of cardiac health [12], or to predict incident atrial fibrillation (AF) [13], or one-year mortality [14]. We contribute by reducing the need for labels, and by focusing on extracting generally expressive representations from ECGs rather than a representation optimized for a single task.

#### Contrastive learning

Contrastive learning is a self-supervised learning method that requires training data only to be labeled with notions of positive pairs (data that go together) and negative pairs (data that are distinct). The SimCLR procedure introduced by [7] shows that contrastive learning yields reduced representations of data that are linearly reusable in new tasks. Many papers have recently experimented with the SimCLR procedure in the medical domain. [5] used the SimCLR procedure in dermatology and X-ray classification tasks. They defined positive pairs as both modified versions of the same image and images from different views of the same skin condition. [15] experimented with SimCLR using many different definitions of a positive pair, including MRI images from different patients that show the same area of the body. [16] defines positive pairs across modalities, between an X-ray image and its associated text report.

A few works utilize the value of subject-specific information by defining positive pairs using non-overlapping segments of temporal signals [17], [18]. These approaches are shown to be especially beneficial in the low-label regime. [6] apply the SimCLR procedure to 12-lead ECGs, defining positive pairs by different leads from the same ECG or as different non-overlapping time segments within a single ECG. They show improved performance in rhythm classification tasks compared to other pre-training strategies in both transfer learning and representation learning regimes. PCLR builds on these works by defining positive pairs across different ascertainments from the same patient rather than segments of the same ascertainment. PCLR does not require segments to be taken from the ECGs, or augmentations that modify the ECGs, which means that the model trains on the unmodified data seen at evaluation time. Furthermore, compared to other ECG contrastive pre-training work, PCLR was trained with millions rather than tens of thousands of ECGs.

[19] apply a different contrastive learning procedure introduced by [20] in lumbar MRIs, but notably define positive pairs in the same way that PCLR does—as pairs of MRIs from the same patient at different times. [20] also make use of image domain specific data augmentations, such as random rotations. We build on this work by demonstrating that with a large dataset of ECGs, a contrastive loss based on patient identity across different ECGs across time is highly performant, expressive, and practical. Unlike all of the other approaches, PCLR does not utilize augmentations and instead relies on the shared underlying biology of different ECGs taken from the same patient.

## Materials and methods

### Ethics statement

The datasets for model training and evaluation was approved by the Institutional Review Board (IRB) at both hospitals from which the data were collected, with a waiver of informed consent.

### PCLR pre-training

PCLR uses a deep residual convolutional neural network to build representations of ECGs. In pre-training, the network learns to build representations of ECGs specific to an individual and is therefore rewarded when representations of different ECGs from the same person are similar. The network is penalized when representations of ECGs from different people are similar. For example, if patient *A* has ECG *x*_*i*_ taken in 1988 and ECG *x*_*j*_ taken in 2001, then (*x*_*i*_, *x*_*j*_) is a positive pair. If patient *B* has ECG *x*_*k*_, then both (*x*_*i*_, *x*_*k*_) and (*x*_*j*_, *x*_*k*_) are negative pairs.

**Fig 3:**
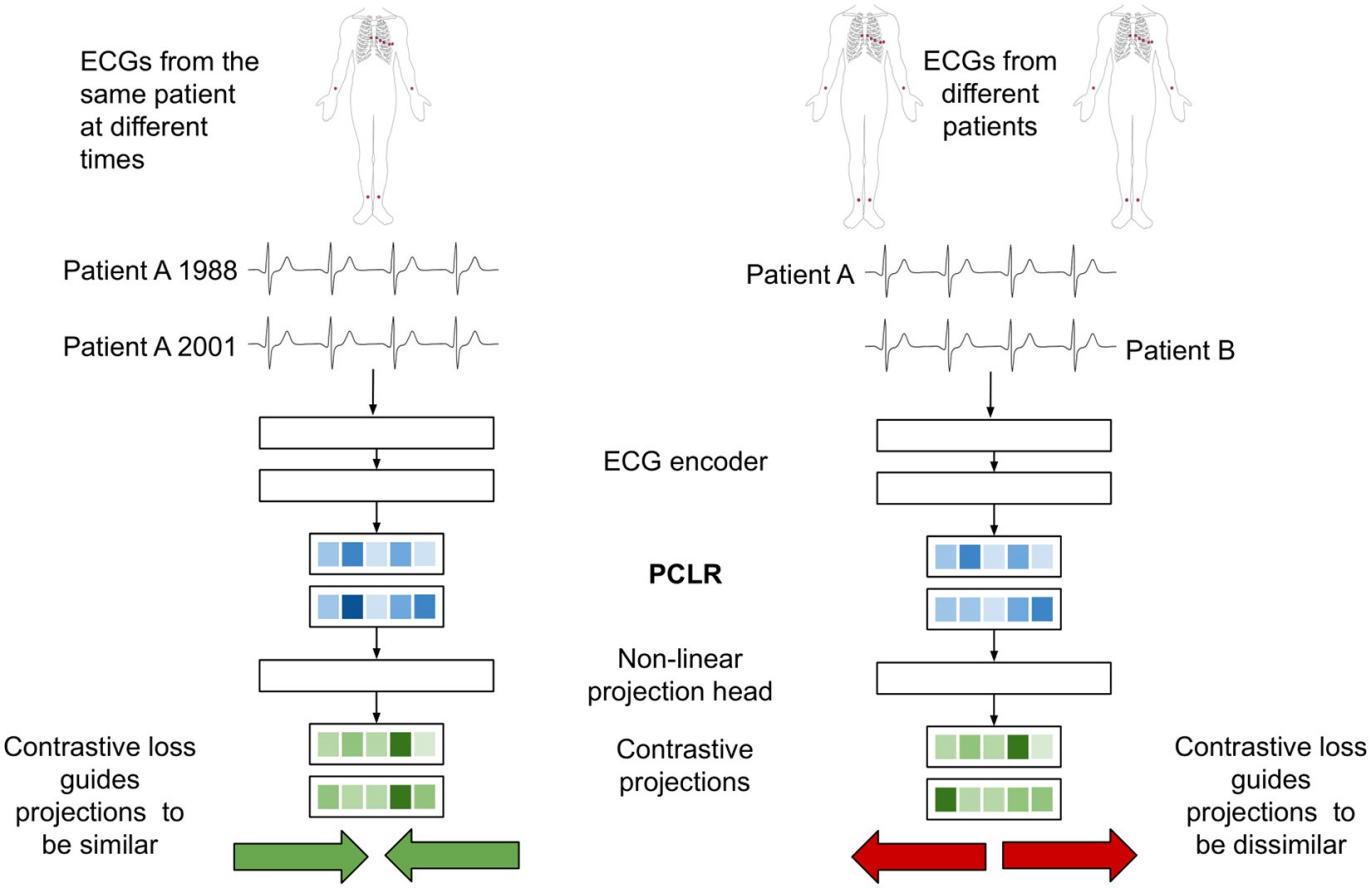
PCLR pre-training procedure for a pair of ECGs from the same patient (left) and from different patients (right).

PCLR pre-training has four components (Fig 3):

1. An *ECG selection module* which selects pairs of ECGs from individuals. For example, it could select ECG *x*_*i*_ from an individual in 1988, and ECG *x*_*j*_ in 2001 from the same participant.
2. An *ECG encoder* *f*(⋅), which produces a compact representation of each 12-lead ECG. Given ECG *x*_*i*_, it outputs the encoding *f*(*x*_*i*_) = *h*_*i*_. When used in linear models, we refer to this representation as PCLR.
3. A *projection head* *g*(⋅), which projects ECG representations into the space where the contrastive loss is applied. For example, *g* could be applied to *h*_*i*_, giving the projection *z*_*i*_ = *g*(*h*_*i*_). [7] showed that pre-training with a non-linear projection head improves the usefulness of the learned representation.
4. A *contrastive loss function* which is used to train the ECG encoder and projection head. The contrastive loss function *ℓ*_*i*,*j*_ is low when the cosine similarity is high between projections of ECGs from the same patient, *z*_*i*_ and *z*_*j*_. *ℓ*_*i*,*j*_ also encourages the cosine similarity to be low between ECGs coming from different patients, e.g. *z*_*i*_ and *z*_*k*_.

#### ECG selection module

Random pairs of ECGs from each individual are selected in every batch (S2 Text) regardless of time or changed health between the ECGs’ acquisitions. We find the random selection approach effective, and leave more advanced strategies to future work.

#### ECG encoder

**Fig 4:**
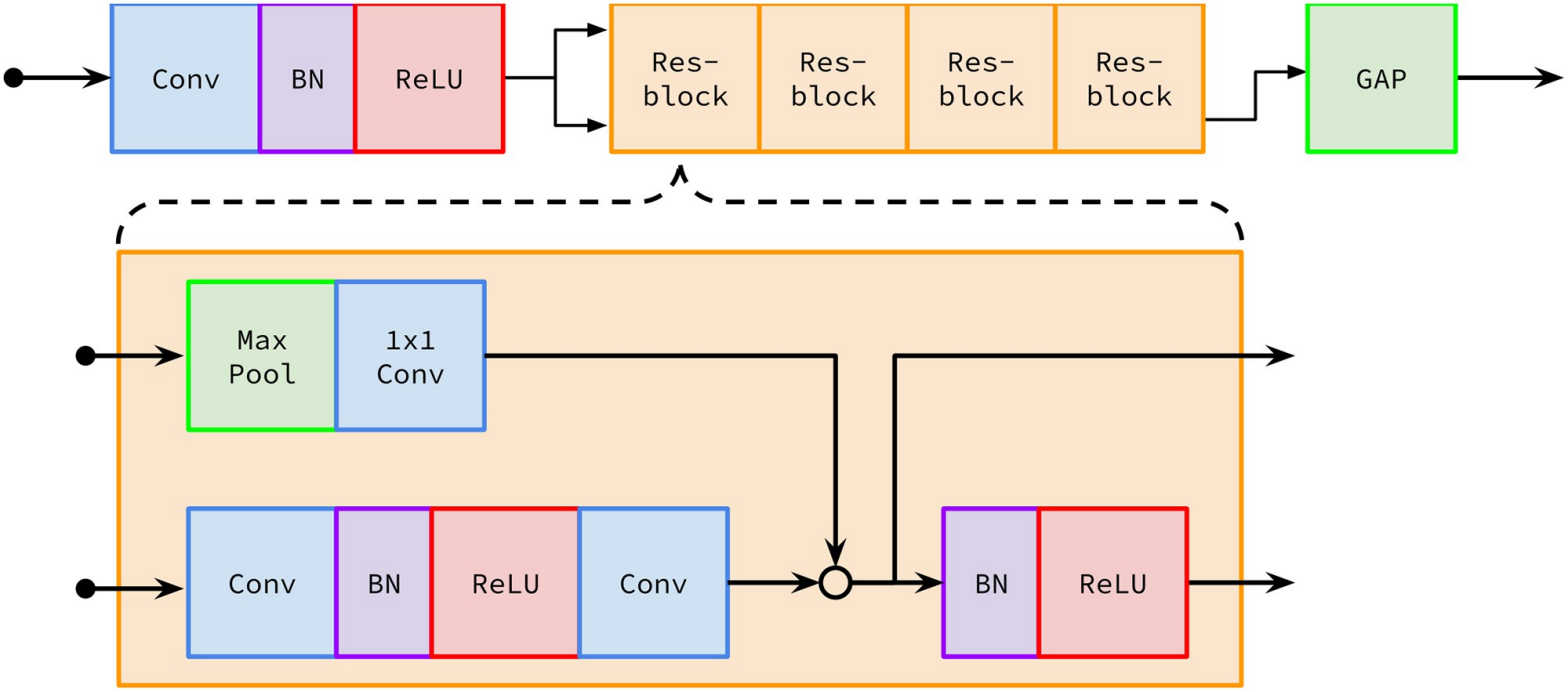
The ECG encoder architecture. Designed by [8] with modifications including global average pooling (GAP). *Conv* means 1D convolution, *BN* means batch normalization, *Max Pool* means 1D max pooling, *ReLU* means rectified linear unit, and *Res-block* means residual block.

To facilitate our comparisons, we used the same encoder architecture developed by [8] (Fig 4). We note that our approach will work with any encoding architecture, not just that of [8]. In order to adapt it to representation learning, one dimensional global average pooling (GAP) [21] was applied to the output of the final residual block in the Ribeiro architecture, yielding a 320-dimensional representation for each ECG. We chose 320 dimensions because it allowed us to adapt and compare against a proven ECG encoder architecture with minimal change. We used the same embedding architecture, and thus the same representation dimension, for all of the pre-training approaches we compared, so we believe that the relative performance of the methods would stay the same at different dimensions.

#### Projection head

**Fig 5:**
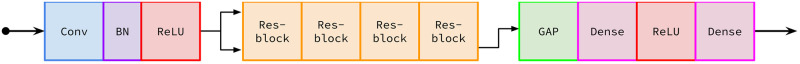
The full architecture used in PCLR pre-training. The projection head, beginning at the first Dense (fully connected) layer, is applied to the output of the ECG encoder.

The projection head follows the encoder and is solely used in pre-training. [7] showed that a non-linear projection head improves the quality of the representations from the encoder. Our projection head was a fully connected layer with 320 units followed by a ReLU activation followed by another 320 unit fully connected layer (Fig 5). The full tensorflow model summary is in S1 Text.

#### Contrastive loss function

The contrastive loss function guides the outputs of the projection head to be similar for ECGs that come from the same patient and distinct for ECGs from different patients. We used the normalized temperature-scaled cross entropy loss with temperature parameter *τ* = 0.1. For a pair of ECGs from the same patient, (*x*_*i*_, *x*_*j*_), a positive pair, the encoder followed by the projection head yield projections *g*(*f*(*x*_*i*_)) = *z*_*i*_ and *g*(*f*(*x*_*j*_)) = *z*_*j*_. Given a batch of *N* patients and letting *sim* denote the cosine similarity yields loss

$$\begin{array}{c} \ell_{i,j}=-\text{log}\frac{\text{exp}\left[\text{sim}\left(z_{i},z_{j}\right)/\tau \right]}{\sum_{k=1}^{2N,k\neq i}\text{exp}[\text{sim}\left(z_{i},z_{k}\right)/\tau ]}. \end{array}$$

In a single minibatch of *N* patients, the loss is computed between all positive pairs. Let *p*_1_ be the index of patient *p*’s first ECG in a minibatch, and *p*_2_ be the index of patient *p*’s second ECG in the minibatch. Then the loss for the batch is

$$\begin{array}{c} L_{\text{batch}}=\sum_{p=1}^{N}\ell_{p_{1},p_{2}}. \end{array}$$

#### PCLR pre-training optimizer details

During pre-training the mini batch size was 1,024 ECGs drawn from 512 patients. The model was trained for 50 epochs using the Adam optimizer [22]. The learning rate started at 0.1 and was decayed every epoch according to a half-period cosine schedule with period 50 epochs [23].

### Applying PCLR to new tasks using linear evaluation

Training linear models on learned representations is known as *linear evaluation*. Linear evaluation has been shown to be a useful indicator of the performance of more complex models trained on learned representations [24]. Furthermore, linear evaluation allows us to test the usefulness of PCLR to practitioners who lack the resources or expertise to train their own neural network and to facilitate integration into existing clinical models (e.g. Cox proportional hazard analyses). We applied linear evaluation to PCLR in a similar procedure to SimCLR:

1. Apply the PCLR-trained ECG encoder to *N* ECGs to get 320 features for each ECG. This results in an *N* × 320 feature vector, which we call PCLR. We then normalize each column by subtracting its mean and dividing by its standard deviation.
2. Train a linear or logistic ridge regression model on PCLR. We use four fold cross validation to select the optimal *ℓ*^2^ penalty from 10 values logarithimcally-evenly spaced between 10^−6^ and 10^5^.
3. Evaluate the linear model on holdout data. We apply the ECG encoder to the holdout ECGs, and normalize the resultant representations using the training summary statistics.

### Cohort

We used ECGs and metadata from two hospitals: Massachusetts General Hospital (MGH) and Brigham and Women’s Hospital (BWH). The data were ingested using the ML4H repository [25]. We pre-trained the ECG encoder using PCLR in a pre-training cohort taken from MGH. In the pre-training cohort, we extracted age, sex, and ECG sampling rate from each ECG. The pre-training cohort is further described in Pre-training cohort from MGH. We built two cohorts from BWH: a set for training supervised models from scratch, and a validation set. The BWH datasets are further described in (BWH cohorts).

#### Choice of evaluation tasks

In order to evaluate PCLR and the baseline methods, four tasks were chosen for their disparate biological bases: left ventricular hypertrophy (LVH) classification, AF classification, sex classification, and age regression. AF is marked on the ECG by an irregular rhythm and missing P-waves. AF has been associated with a greatly increased risk of stroke [26]. LVH is defined by an increase in left ventricular mass. LVH can be detected from the ECG using voltage criteria with high specificity but low sensitivity [27]. LVH is associated with an increased risk of cardiovascular diseases and death [28]. Age and sex detection have been shown to be possible from the ECG by works including [12]. Taken together, age regression, sex determination, and AF and LVH detection serve as useful benchmarks because prior work have leveraged the ECG for these predictive tasks. Moreover, the prediction of AF, by itself, is clinically important as its detection has important clinical consequences.

#### Data extraction

In both the pre-training and test cohorts, age, sex, heart rate, PR interval, QRS duration, and QT interval are all reported as tabular fields. LVH and AF were defined using a free text diagnosis field by checking for containment of keywords. The ECG waveforms in both cohorts are recorded for ten seconds at either 250 Hz or 500 Hz with amplitudes represented in microvolts as 16 bit integers. All 12 leads of the ECG are recorded in their own fields. As preparation for training the ECG encoder, we divided the amplitudes for each lead by 1,000 to get units of millivolts, convert to 32 bit floats, and then use linear interpolation to fit each lead into 4,096 samples. Once each lead has been interpolated to the same length, we them into a 4, 096 × 12 matrix with lead order {I, II, III, AVR, AVL, AVF, V1, V2, V3, V4, V5, V6}. The ECG pre-processing code is available at https://github.com/broadinstitute/ml4h/tree/master/model_zoo/PCLR.

Our overarching goal is to develop a method that would be easy for any health care provider to use. As routine electrocardiograms can contain noise that typically arises from baseline wander and muscle artifact [29], we strove to develop a method that would be robust to these noise sources. We therefore chose to not pre-process the ECG signals to remove artifacts that may be seen during routine electrocardiography. Given the size of our pre-training dataset (which contains several million ECGs), this approach should help the model learn to be robust to noise.

#### Pre-training cohort from MGH

Patients with only one ECG were filtered out in order to make the PCLR contrastive loss more informative. That leaves 404,929 patients with 3,229,408 ECGs. 90% of the MGH patients were selected into a training set and the remaining 10% of patients in the validation set. The summary statistics of the pre-training cohort are shown in S2 Appendix. The validation set is used to pick the checkpoint of the model with the best performance during training.

#### BWH cohorts

We produced a test set from BWH (B-test) with 10,000 ECGs, and a series of training sets of increasing size: 640, 1280, 2560, 5120, 10240, and 20480 sample. We called these B-640, B-1280, etc. Each of the B-series is a subset of the one larger than it. For example, B-1280 is a subset of B-2560. Not all features were defined in the presence of AF, so separate AF datasets (AF-640 through AF-20480) were created. Summary statistics and selection criteria for all BWH cohorts are shown in S2 Appendix.

## Results

### PCLR linear evaluation compared to training from scratch

**Fig 6:**
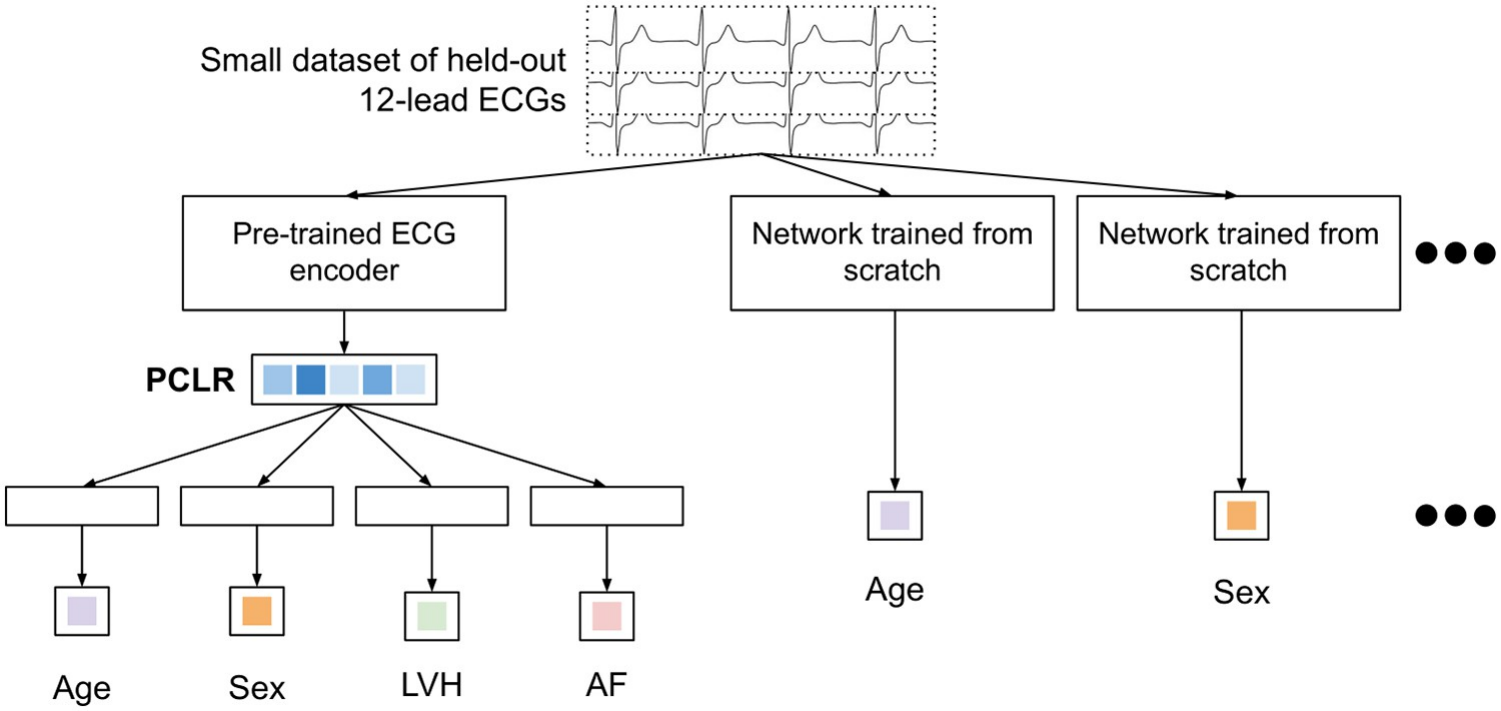
Flowchart comparing training models on PCLR and training models from scratch.

We trained PCLR on ECG representations extracted from B-640 through B-20480 to regress age and classify sex, LVH, and AF (AF on AF-640 through AF-20480). Performance was evaluated on B-test and AF-test. We built and trained a neural network with randomly initialized weights for each of the four tasks and six training dataset sizes. To make the comparison fair, we used the same ECG encoder as we used to train PCLR (Fig 4) followed by a linear fully connected layer. Our overall approach is outlined in (Fig 6). In each classification task, the final layer outputs two values and the model was trained using the categorical cross entropy. In age regression, the linear layer outputs one value and the models were trained using the mean squared error. For each task and each number of training labels, a grid search over learning rates in {10^−2^, 10^−3^, 10^−4^} and dropout rates on the convolutional layers in {0, 0.1, 0.2} was used to select the best model. All of the models were trained using the Adam optimizer until the validation loss stops improving for five epochs, taking the checkpoint of the network with the lowest validation loss for evaluation.

**Fig 7:**
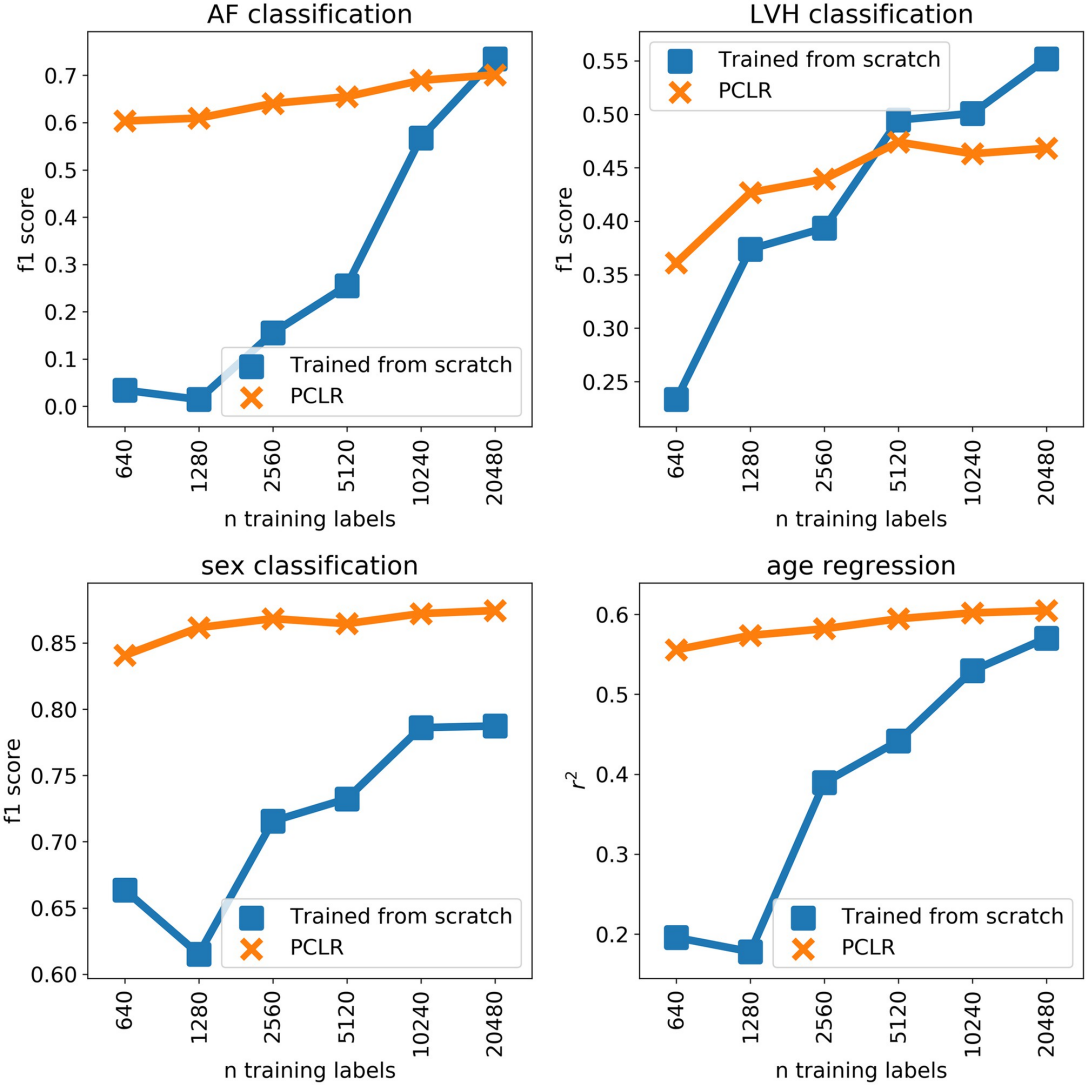
Results of training from scratch vs. linear models trained on PCLR.

We found PCLR is better than a model trained from scratch for all four tasks we evaluated up to at least 5,000 labeled training examples (Fig 7). PCLR was especially effective at sex classification, which may be because sex is a patient specific property that, in our population, is typically the same for all ECGs corresponding to a given patient. The connection between patient contrastive learning and the performance on the four tasks is further explored in the Comparison to standard ECG features section. The fact that PCLR was able to perform well for LVH classification (found in four percent of ECGs) and AF classification (found in five percent of ECGs) indicates that PCLR can be useful in the presence of class imbalance.

### PCLR compared to other pre-training approaches

We compared PCLR to three other approaches for extracting ECG representations. All three comparison approaches trained on the same ECGs. The comparison models used the same ECG encoder as in PCLR and thus also yielded 320 dimensional representations. These experiments were designed to compare the effects of different pre-training objectives on the usefulness of the ECG representations.

#### Ribeiro ECG encoder

Ribeiro et al. [8] trained a model to classify first degree AV block, right bundle branch block, left bundle branch block, sinus bradycardia, atrial fibrillation, and sinus tachycardia using 2,322,513 ECG records from 1,676,384 different patients. We attempted to directly use the weights released by [8] and to train our own model with the same architecture on data from MGH using the same six classification targets (details in S1 Appendix). In the following experiments, the results of our version of the Ribeiro model are reported, since it was uniformly better. This is likely because the MGH population is more similar to the BWH population than the patient population used in [8]. We label linear evaluation of ECG representations from the encoder with Ribeiro weights Ribeiro-R.

#### Convolutional auto encoder (CAE)

Auto Encoders are a standard approach to extracting compact representations of data. Others have found success applying CAEs to ECG data for compression [30] and for pre-training for arrhythmia detection [31]. We built a CAE using the ECG encoder by applying a series of transpose convolutions to the output of the ECG encoder (architecture details in S3 Text). This approach led to faithful ECG reconstructions (optimizer details and pre-training results in S1 Appendix).

#### Contrastive learning of cardiac signals (CLOCS)

A class of methods based on SimCLR to pre-train ECG models using ECG domain knowledge was developed by [6]. We trained the same pre-training architecture used for PCLR with the approach [6] found most effective, contrastive multi-segment multi-lead coding (details in S1 Appendix). Contrastive multi-segment multi-lead coding defines positive pairs as different sub-segments and different leads taken from the same ECG. By comparing against CLOCS, we showed the trade offs of picking contrastive positive and negative pairs based on patient identity.

#### Pre-training approaches comparison results

We applied linear evaluation to the output of the encoder portion of PCLR and the three comparison models. The linear models were trained on all BWH training sets, and evaluated on the BWH test set. PCLR had the best or tied for the best performance in all tasks besides AF classification across all training dataset sizes (Table 1). The particularly poor performance of Ribeiro-R in LVH classification could be because LVH is read from ECGs using voltage amplitudes [27], which may not be necessary to learn for rhythm classification. Similarly, CLOCS was designed with rhythm classification in mind, and it may be enough to distinguish whether leads belong to the same ECG or come from different subsections of the same ECG based on rhythm.

**Table 1:** Performance metrics of the ECG encoder methods.

task	n training labels	Ribeiro-R	CAE	CLOCS	PCLR
LVH (f1 score)	640	0.14 ± 0.02	0.25 ± 0.02	0.19 ± 0.02	**0.36 ± 0.02**
	1280	0.19 ± 0.02	0.37 ± 0.02	0.18 ± 0.02	**0.43 ± 0.02**
	2560	0.27 ± 0.03	0.33 ± 0.02	0.19 ± 0.02	**0.44 ± 0.02**
	5120	0.28 ± 0.02	0.41 ± 0.03	0.20 ± 0.02	**0.47 ± 0.02**
	10240	0.33 ± 0.03	0.43 ± 0.03	0.23 ± 0.02	**0.46 ± 0.03**
	20480	0.29 ± 0.03	**0.49 ± 0.03**	0.27 ± 0.03	0.47 ± 0.03
age (*r*^2^)	640	0.33 ± 0.01	0.24 ± 0.01	0.41 ± 0.01	**0.56 ± 0.01**
	1280	0.36 ± 0.01	0.29 ± 0.01	0.43 ± 0.01	**0.57 ± 0.01**
	2560	0.38 ± 0.01	0.32 ± 0.01	0.47 ± 0.01	**0.58 ± 0.01**
	5120	0.40 ± 0.01	0.34 ± 0.01	0.49 ± 0.01	**0.59 ± 0.01**
	10240	0.41 ± 0.01	0.35 ± 0.01	0.50 ± 0.01	**0.60 ± 0.01**
	20480	0.42 ± 0.01	0.36 ± 0.01	0.51 ± 0.01	**0.60 ± 0.01**
sex (f1 score)	640	0.72 ± 0.00	0.77 ± 0.00	0.77 ± 0.00	**0.84 ± 0.00**
	1280	0.74 ± 0.00	0.78 ± 0.00	0.78 ± 0.00	**0.86 ± 0.00**
	2560	0.75 ± 0.00	0.79 ± 0.00	0.78 ± 0.00	**0.87 ± 0.00**
	5120	0.76 ± 0.00	0.80 ± 0.00	0.78 ± 0.00	**0.86 ± 0.00**
	10240	0.77 ± 0.00	0.80 ± 0.00	0.79 ± 0.00	**0.87 ± 0.00**
	20480	0.77 ± 0.00	0.80 ± 0.00	0.80 ± 0.00	**0.87 ± 0.00**
AF (f1 score)	640	n/a	0.20 ± 0.02	**0.68 ± 0.02**	0.60 ± 0.02
	1280	n/a	0.23 ± 0.02	**0.76 ± 0.01**	0.61 ± 0.02
	2560	n/a	0.24 ± 0.02	**0.78 ± 0.01**	0.64 ± 0.02
	5120	n/a	0.28 ± 0.02	**0.79 ± 0.01**	0.65 ± 0.02
	10240	n/a	0.26 ± 0.02	**0.79 ± 0.01**	0.69 ± 0.02
	20480	n/a	0.24 ± 0.02	**0.80 ± 0.01**	0.70 ± 0.02

Bold results indicate the best model with a fixed task and number of training labels. The best models are picked using 1,000 bootstraps of the test data. The performance ranges are ± one standard deviation of the bootstrapped performances.

### Comparison to standard ECG features

**Fig 8:**
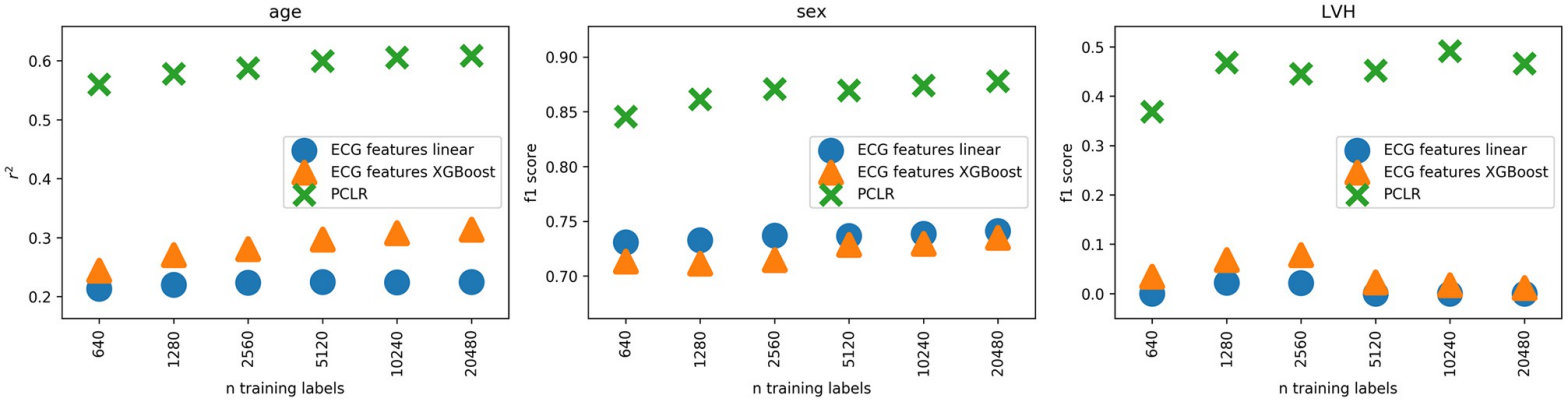
Comparison against standard ECG features.

We compared using PCLR to using seven standard features extracted from the ECG: HR, PR interval, QRS duration, QT interval, P-axis, R-axis, and T-axis, which is another default approach for a clinician researcher. The comparison was made in LVH and sex classification and age regression. We cannot compare on AF classification, because some of the ECG features are undefined in AF. We trained linear and XGBoost [32] models on the seven ECG features on all BWH training sets, and evaluated on the BWH test set. The linear model hyperparameters were optimized using the same grid search over a range of regularization strengths as the PCLR linear models. The XGBoost hyperparameters were selected using grid search over maximum depths of 2, 4, and 6, and numbers of estimators of 50, 100, and 200. Across all three tasks, PCLR was substantially better (Fig 8). These results show the advantage of using learned representations over hand designed features.

## Discussion

We compared PCLR to many of the options available for ECG modeling when there are few training labels: training a new model from scratch, using hand designed features of the ECG, and pre-training models with different pre-training objectives. By comparing across four different tasks with different biological underpinnings, we evaluated the generalizability of the approaches evaluated. PCLR yielded large performance increases compared to training from scratch with limited training data across all four tasks, which demonstrates the relevance of the patient contrastive learning objective for ECG modeling. PCLR was also almost always the best option for pre-training and had the most consistent performance across the four tasks. The results show that PCLR is a generalist approach which can be applied without needing finetuning. Furthermore, PCLR was designed to only require linear models to use for new tasks. By using the model weights we released, the computational cost of PCLR is running inference and then training linear models, which is significantly cheaper than training a model from scratch.

We can use the framework for understanding contrastive learning presented in [33] to understand the effectiveness of PCLR. To make contrastive approaches as effective as possible, pairs, pairs for the contrastive learning task should be chosen so that each element of the pair shares features relevant to the downstream task, and does not share irrelevant features. They call this the “InfoMin” principle. In the PCLR pre-training objective, the features that are generalizably shared between pairs of ECGs are features realted to sex, genetics, and slowly progressing health states. The InfoMin principle thus explains why PCLR representations were effective for sex and LVH classification, which tend to be stable over time. Age also is on average not very different between patients’ ECGs, which were taken on average 262 days apart in the training data. PCLR’s AF detection performance may be partially explained by the genetic features which contribute to AF [34]. On the other hand, PCLR may have a disadvantage at detecting AF in patients with paroxysmal AF, since the presence of AF will be inconsistent in ECGs from such patients.

## Conclusion

The application of deep learning to clinical datasets that have a few labeled training examples raises a number of issues. Fruitful application of neural networks to these data often requires the use of additional methods to mitigate the effects of small sample sizes. Consequently, the application of deep learning in the regime of limited training data in health care has required researchers with both in depth knowledge of machine learning and deep domain specific knowledge. Unfortunately, however, few researchers have both.

Here we developed a contrastive learning approach, PCLR, which helps resolve this gap. PCLR corresponds to a relatively low dimensional representation of high dimensional 12-lead ECG data. A key aspect of the approach is that these representations are constructed in a manner to ensure that different ECGs, which arise from the same patient, are more similar to one another relative to ECG-representations arising from different patients. This is a unique-to-health-care self-supervised approach for building useful representation of rich medical data. We demonstrated that PCLR is performant, expressive, and practical for clinical researchers to use across a variety of tasks. We also outlined the regimes where PCLR is the most performant approach to adopt.

The success of PCLR suggests that building patient-centered representations of multi-modal health care data, not just ECGs, is an important direction of future research. For example, one could consider merging the patient identity component of PCLR with the joint echo-cardiogram-text modeling of [16]. Such an approach could lead to unified representations of patients’ heart health from echo-cardiograms, ECGs, and other cardiology data modalities.

## Supporting information

S1 Text PCLR pre-training model architecture. The text was produced using the tensorflow 2.3 keras model summary function. All activations are ReLU, and all convolutions have filter size of 16.
(TXT)

S2 Text PCLR minibatch pre-training procedure in Python 3.6. (PY)

S3 Text CAE architecture. The text was produced using the tensorflow 2.3 keras model summary function.
(TXT)

S1 Appendix Pre-training details of PCLR, Ribeiro model, CLOCS model, and CAE model. (PDF)

S2 Appendix Cohort selection processes and characteristics of MGH and BWH. (PDF)

S1 Fig Reconstruction of lead I of an ECG by the CAE. The ECG shown was randomly selected from the MGH validation data.
(TIFF)

S1 Data Every model’s predictions and the truth values of the test data. Fig 7, Table 1, and Fig 8 were produced using S1_Data.csv, which contains predictions and truth labels for each model and task.
(CSV)
