# Supplementary material for: Patient contrastive learning: A performant, expressive, and practical approach to electrocardiogram modeling
Source: PLoS Comput Biol. 2022 Feb 14;18(2):e1009862. doi: 10.1371/journal.pcbi.1009862 (PMC8880931; doi:10.1371/journal.pcbi.1009862)
Supplement: S1 Appendix — (PDF) [file pcbi.1009862.s004.pdf]

Pre-training appendix to: Patient contrastive learning: a performant, expressive, and practical approach to electrocardiogram modeling

Nathaniel Diamant[1,2], Erik Reinertsen[1,3], Steven Song[3], Aaron Aguirre[3, 4, 5], Collin Stultz[1, 3, 6, 7], Puneet Batra[2*],

**1** Research Laboratory of Electronics, MIT, Cambridge, MA
**2** Data Sciences Platform, Broad Institute of MIT and Harvard, Cambridge, MA
**3** Division of Cardiology, Massachusetts General Hospital, Boston, MA
**4** Center for Systems Biology, Massachusetts General Hospital Research Institute and Harvard Medical School, Boston, MA
**5** Wellman Center for Photomedicine, Massachusetts General Hospital Research Institute and Harvard Medical School, Boston, MA
**6** Department of Electrical Engineering and Computer Science, MIT, Cambridge, MA
**7** Harvard-MIT Division of Health Sciences and Technology, Cambridge, MA

* pbatra@broadinstitute.org

# Contents

**1 PCLR pre-training metrics** 1

**2 Ribeiro model pre-training** 2

**3 CLOCS pre-training** 3

**4 CAE pre-training** 4

# 1 PCLR pre-training metrics

The validation temperature-scaled cross entropy loss with temperature parameter $\tau = 0.1$ was 1.46. The validation accuracy at identifying whether a pair of ECGs came from the same patient out of 512 patients was 94.0% in an average batch. Note that the 94.0% accuracy includes identification of some ECGs as identical to themselves due to the random ECG selection procedure.

## 2 Ribeiro model pre-training

The Ribeiro model was trained on the same training ECGs as the PCLR model with the same optimizer settings, except that the learning rate was set to $10^{-2}$ and dropout on the convolutional layers was set to 0.2 based on a grid search. Labels for the six classes were derived from a diagnosis text field associated with the ECGs using simple regular expressions. The Ribeiro model's validation metrics are shown in Table 1.

Table 1: Performance metrics of Ribeiro architecture trained on MGH data

| | validation prevalence | f1 score | AUCROC |
|---|---|---|---|
| right bundle branch block | 9.17% | 0.76 | 0.97 |
| left bundle branch block | 3.20% | 0.77 | 0.99 |
| first degree AV block | 6.72% | 0.71 | 0.98 |
| atrial fibrillation | 8.11% | 0.81 | 0.97 |
| sinus bradycardia | 13.06% | 0.85 | 0.98 |
| sinus tachycardia | 7.48% | 0.83 | 0.98 |

# 3 CLOCS pre-training

The CLOCS model introduced in [1] was trained using the CMSMLC procedure, with which the CLOCS authors got the best results. In our implementation of the CMLSMLC procedure, a positive pair was defined by an ECG modified in two ways. One half of the pair had either the first or second five seconds of the ECG set to zero. It also had a random selection of ten of the 12 leads set to zero. The other half of the positive pair had the other five seconds of the ECG set to zero and another random selection of 10 leads set to zero. The model was trained with the same optimizer settings as in PCLR, except with a lower learning rate of $10^{-2}$ selected by a grid search over learning rates of validation loss. The validation temperature-scaled cross entropy loss with temperature parameter $\tau = 0.1$ was 1.83. The validation accuracy at identifying whether a pair of perturbed ECGs came from the same patient out of 512 patients was 88.0%.

# 4 CAE pre-training

The CAE was trained with the same optimizer as PCLR, except with an initial learning rate of $10^{-4}$. The loss function was logcosh, which led faster convergence than experiments with mean squared error (MSE). The validation logcosh was 0.0037 and the validation MSE was 0.0221. The final reconstructions were highly accurate. The CAE architecture uses the same architecture as the ECG encoder, followed by a fully connected later and a series of one-dimensional transpose convolutions with batch normalization and the swish activation [2].

## References

[1] Kiyasseh D, Zhu T, Clifton DA. CLOCS: Contrastive Learning of Cardiac Signals across Space, Time, and Patients. In: Meila M, Zhang T, editors. Proceedings of the 38th International Conference on Machine Learning. vol. 139 of Proceedings of Machine Learning Research. PMLR; 2021. p. 5606–5615.

[2] Ramachandran P, Zoph B, Le QV. Searching for Activation Functions. In: 6th International Conference on Learning Representations, ICLR 2018, Vancouver, BC, Canada, April 30 - May 3, 2018, Workshop Track Proceedings. OpenReview.net; 2018.
