# Supplementary material for: Patient contrastive learning: A performant, expressive, and practical approach to electrocardiogram modeling
Source: PLoS Comput Biol. 2022 Feb 14;18(2):e1009862. doi: 10.1371/journal.pcbi.1009862 (PMC8880931; doi:10.1371/journal.pcbi.1009862)
Supplement: S2 Appendix — (PDF) [file pcbi.1009862.s005.pdf]

Cohort description appendix to: Patient contrastive learning: a performant, expressive, and practical approach to electrocardiogram modeling

Nathaniel Diamant[1,2], Erik Reinertsen[1,3], Steven Song[3], Aaron Aguirre[3, 4, 5], Collin Stultz[1, 3, 6, 7], Puneet Batra[2*],

1 Research Laboratory of Electronics, MIT, Cambridge, MA
2 Data Sciences Platform, Broad Institute of MIT and Harvard, Cambridge, MA
3 Division of Cardiology, Massachusetts General Hospital, Boston, MA
4 Center for Systems Biology, Massachusetts General Hospital Research Institute and Harvard Medical School, Boston, MA
5 Wellman Center for Photomedicine, Massachusetts General Hospital Research Institute and Harvard Medical School, Boston, MA
6 Department of Electrical Engineering and Computer Science, MIT, Cambridge, MA
7 Harvard-MIT Division of Health Sciences and Technology, Cambridge, MA

* pbatra@broadinstitute.org

Contents

1 MGH cohort characteristics **2**

2 BWH selection criteria **3**

3 BWH cohort characteristics **4**

4 BWH AF cohort characteristics **5**

1 MGH cohort characteristics

	training	validation
Total Patients	364,436	40,493
Total ECGs	2,907,705	321,703
ECGs/patient (median; mean)	4.0; 7.98 ± 11.7	4.0; 7.94 ± 11.7
Time between ECGs days (median; mean)	76; 262 ± 467	75; 261 ± 467
Age (median; mean)	64.0; 62.2 ± 16.9	64.0; 62.1 ± 17.1
Sex (Male : Female)	192,072 : 169,633	21,278 : 18,894
Sampling rate (250Hz : 500 Hz)	1,939,279 : 968,426	214,476 : 107,227
Taken in ICU	9.11%	9.16%
Taken in ED	11.99%	12.20%

2 BWH selection criteria

1. Require that the patient has no ECGs recorded in MGH
2. Take only the most recent ECG from each patient
3. Restrict age to between 20 and 90 years
4. Require a maximum absolute voltage amplitude of 10 millivolts
5. Require the presence of age, sex, diagnosis text, heart rate, PR interval, QT interval, QRS duration, P-axis, R-axis, and T-axis from each ECG
6. P-axis must not be -1 degrees, because that was used as an indicator of missingness by the automated software

BWH cohort characteristics are shown in Section 3. When AF is present in an ECG, the P-wave is missing, so the PR interval is not defined. For that reason, we produced additional versions of each BWH dataset, AF-test with 10,000 ECGs, AF-640, AF-1280, etc. The selection criteria for ECGs in the AF datasets are the same as the criteria for the non-AF datasets, except we only required the presence of age, sex, heart rate, and diagnosis text from each ECG. Cohort characteristics of AF-20480 and AF-test are shown in Section 4.

3 BWH cohort characteristics

	B-20480			B-test		
continuous feature	median	mean	std	median	mean	std
age	38.14	35.50	26.90	39.71	38.00	27.39
HR	72.00	75.19	17.07	73.00	75.84	17.84
PR	54.76	55.95	12.10	54.76	56.09	12.57
QRS	44.00	45.54	8.83	44.00	46.30	10.14
QT	89.26	89.30	11.52	89.26	89.47	12.11
R-axis	32.00	29.90	41.49	31.00	29.21	44.92
T-axis	42.00	44.81	39.41	42.00	46.83	44.82
P-axis	52.00	50.02	23.66	51.00	48.63	25.39
binary feature	%			%		
Female	55.49			54.13		
Has LVH	3.97			3.95		
Sample rate 250 Hz (otherwise 500 Hz)	46.41			47.21		
Taken in ICU	2.22			2.03		
Taken in ED	26.96			27.44		

4 BWH AF cohort characteristics

	AF-20480			AF-test		
continuous feature	median	mean	std	median	mean	std
age	39.71	37.51	27.34	39.71	38.00	27.39
HR	73.00	76.01	17.94	73.00	75.84	17.84
binary feature	%			%		
Has AF	5.27			5.24		
Female	54.79			54.13		
Sample rate 250 Hz (otherwise 500 Hz)	46.59			47.21		
Taken in ICU	2.56			2.36		
Taken in ED	26.50			25.23		
