# Supplementary figures and images for: Patient contrastive learning: A performant, expressive, and practical approach to electrocardiogram modeling
Source: PLoS Comput Biol. 2022 Feb 14;18(2):e1009862. doi: 10.1371/journal.pcbi.1009862 (PMC8880931; doi:10.1371/journal.pcbi.1009862)

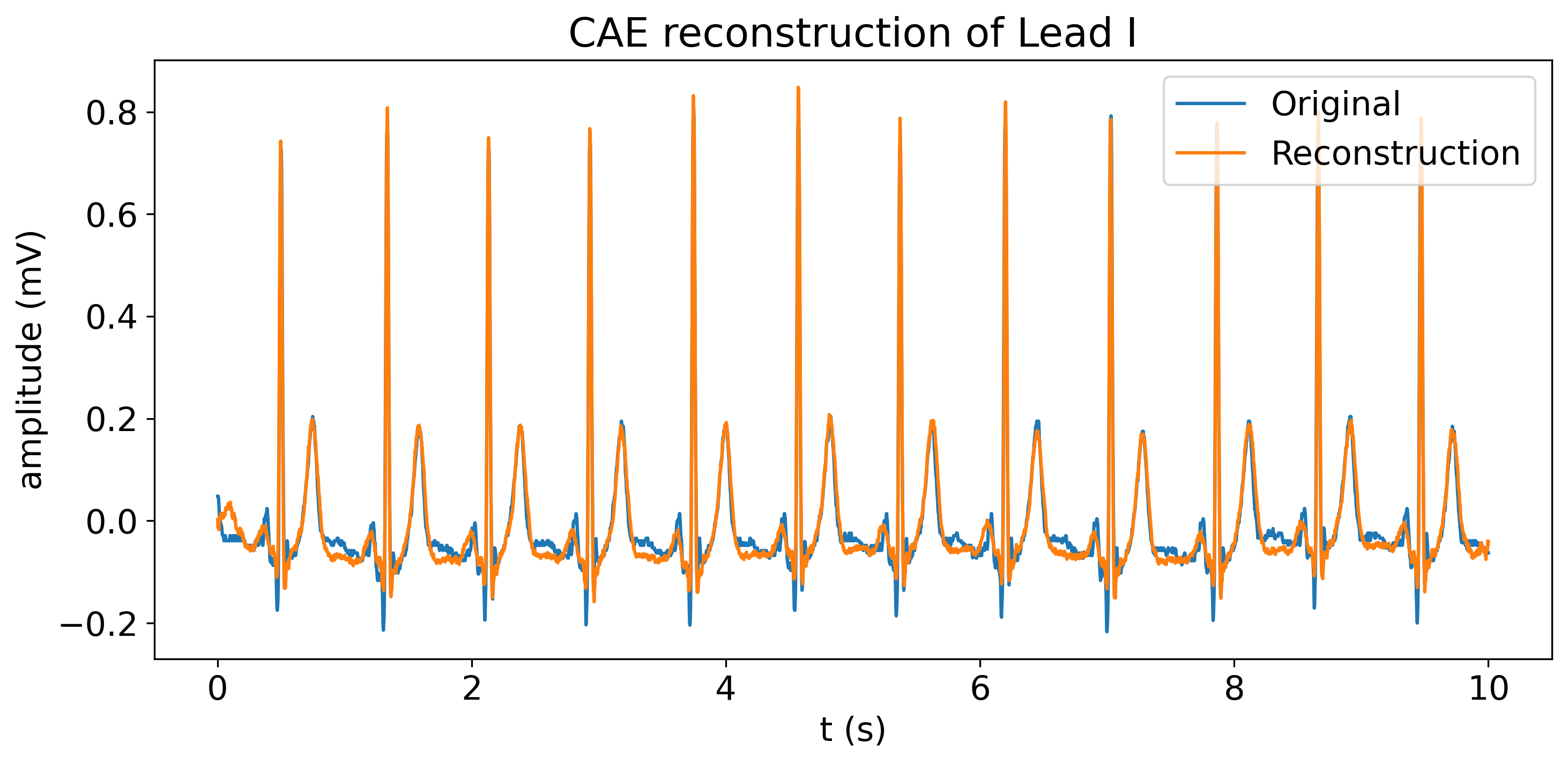

Supplement: S1 Fig — The ECG shown was randomly selected from the MGH validation data. (TIFF) [file pcbi.1009862.s006.tiff]
